# Comparing Loose Clothing-Mounted Sensors with Body-Mounted Sensors in the Analysis of Walking

**DOI:** 10.3390/s22176605

**Published:** 2022-09-01

**Authors:** Udeni Jayasinghe, Faustina Hwang, William S. Harwin

**Affiliations:** 1 Biomedical Engineering, School of Biological Sciences, University of Reading, Reading RG6 6DH, UK; 2 Information Systems Engineering, University of Colombo School of Computing, Colombo 00700, Sri Lanka

**Keywords:** accelerometer, body-mounted sensors, clothing-mounted sensors, gait cycle, healthcare, human movement, IMU, sensor to vertical angle, wearable devices

## Abstract

A person’s walking pattern can reveal important information about their health. Mounting multiple sensors onto loose clothing potentially offers a comfortable way of collecting data about walking and other human movement. This research investigates how well the data from three sensors mounted on the lateral side of clothing (on a pair of trousers near the waist, upper thigh and lower shank) correlate with the data from sensors mounted on the frontal side of the body. Data collected from three participants (two male, one female) for two days were analysed. Gait cycles were extracted based on features in the lower-shank accelerometry and analysed in terms of sensor-to-vertical angles (SVA). The correlations in SVA between the clothing- and body-mounted sensor pairs were analysed. Correlation coefficients above 0.76 were found for the waist sensor pairs, while the thigh and lower-shank sensor pairs had correlations above 0.90. The cyclical nature of gait cycles was evident in the clothing data, and it was possible to distinguish the stance and swing phases of walking based on features in the clothing data. Furthermore, simultaneously recording data from the waist, thigh, and shank was helpful in capturing the movement of the whole leg.

## 1. Introduction

Gait analysis can give a good indication about one’s health, as walking is a common physical activity for many people, performed everyday, and it highly depends on the central nervous system [1,2]. Hence, if there is any functional disorder in the central nervous system, it is reflected in gait patterns [3]. The way gait could be affected by nervous system disorders can be seen with dementia patients and people with Parkinson’s [4,5]. Furthermore, even healthy older adults with slow gait speed may experience deterioration in cognitive function [4]. Hence, monitoring gait patterns could be a method for the early diagnosis of musculoskeletal and central nervous system disorders [3].

Gait analysis technologies can broadly be divided into two categories, non-wearable sensor and wearable sensor technologies [6]. Usually, non-wearable technologies, such as camera-based systems and ground reaction force sensors (GRF), are used in laboratory settings, whereas wearable sensor technologies, such as accelerometers, gyroscopic sensors, magnetometers, force sensors, extensometers, goniometers, active markers, electromyography, sensing fabrics and smartphones, can be used outside and inside the laboratory setting [6]. However, due to having features such as usability, portability, low cost, low power consumption, high sensitivity and small sizes, wearable sensors are commonly used in gait and other movement analyses research [1,6]. In gait research, wearable sensors have been mounted on feet/shoes [7,8,9,10,11], knees, thighs [12], ankles [5,13], shanks [14,15,16], chests [13] and waists [13,17] to analyse the gait parameters such as stride velocity, stride length, step length, cadence, step width, step angle, step time, swing time, stance time, gait phase, joint angles and momentum [6].

In many research studies, gait parameters are extracted from multiple places, such as the hip, knee and ankle joints, and researchers investigate combining the data collected from the different sites [18] Furthermore, other studies have stated how results could be enhanced by using multiple sensors in gait analysis and gait classification [19,20]. Gao et al. have identified the benefits of using multiple sensors with light-weight algorithms as compared with using single wearable sensors with computationally demanding processing to train complex classifiers [21].

However, wearing multiple sensors on a long-term basis could be a cumbersome task for the wearer. Moreover, if the sensors are not reliably worn in the same place in the same orientation every time, the data would not be consistent, which makes the data analysis process more complex. Clothing-mounted sensors and smart garments could potentially address these issues. An important step toward the use of clothing-mounted sensors for gait analysis and/or long-term monitoring, however, is to verify the reliability of the data, for example, by validating the data from clothing-mounted sensors against those from body-mounted sensors. There are only a limited number of research studies on this topic. One such study validated the association between readings from sensors mounted in tight-fitting clothing and sensors mounted on the body with respect to a single activity (dead-lifting) [22]. Another work showed that sensors attached onto fabric produced better signal variations compared with sensors attached with accessories (e.g., bands) that could be used in activity classification [23]. There are research studies conducted with loose clothing-mounted sensors in investigating movement and fall detection [24,25,26]. However, it was noted that these clothing data have not been validated/compared with body-mounted sensor data with different activities. As such, there is a need for a systematic analysis to assess the extent to which everyday wear clothing-mounted sensor data can be used in movement analysis and activity classification.

In our previous work [27] conducted with Actigraph (https://actigraphcorp.com, accessed on 15 November 2019) sensors, the correlations between clothing- and body-mounted sensors in dynamic activities were found to be lower than the correlations observed during static activities such as standing and sitting, due to the sensors being a bit heavy (19 g). Hence, the present study investigates the use of light-weight sensors in the clothing, specifically comparing how well the data from clothing correlate with data from sensors mounted on the frontal side of the lower body in gait analysis. In contrast with studies involving tight garments [13,28], in this study, we investigated everyday loose clothing, as this is likely to be more comfortable and acceptable for wearers.

## 2. Background

A gait cycle consists of two main phases (stance and swing phases), and they are further categorised into eight sub-phases [11,29,30]. Stance phase makes up approximately 60% of a gait cycle, and it starts from initial contact (IC)/heel strike (HS), followed by loading response (foot flat), mid-stance, terminal stance and toe-off (TO) [11,29,30]. The remaining 40% of the gait cycle consists of the initial swing, mid-swing (MS) and terminal swing.

A complete gait cycle is considered to be from one IC/HS to the next IC/HS with the same foot [11,29,30,31,32]. A number of studies have used gyroscope data to track ‘HS’ and ‘TO’ in gait cycles from foot/shoe-mounted sensors [8,9,16,29,33] while others have used either accelerometry alone or both gyroscope and accelerometer data combined. With accelerometer data, MS [12], HS [10] and terminal contact (foot-off) [14] can be used to track gait cycles. When using both accelerometer and gyroscope data, HS, TO, MS and terminal stance can be used to track gait cycles [5,7].

The present study focuses on examining sensor-to-vertical angles as a measure of the sensor’s orientation in space relative to vertical. As a first step, the orientation of each sensor had to be calculated. There are many ways of estimating the orientation of inertial measurement units (IMUs) and magnetic, angular rate, and gravity (MARG) sensors. While an IMU (combination of an accelerometer and a gyroscope) can estimate attitude with respect to the direction of gravity, MARG sensors can estimate orientation considering both the direction of gravity and the magnetic field of the earth [34]. Further, as IMU data are susceptible to noise, sensor fusion techniques have been proposed as a reliable way to estimate orientation [35]. Three possible ways to represent orientation are Euler angles, quaternions and direction cosine matrices (DCM) [35]. In this study, we express sensor orientation as quaternions. Commonly used algorithms that estimate orientation with quaternions as outputs are described by Mahony et al. [36], Madgwick et al. [37] and Sabatini [38]. For this study, the algorithm implemented by Madgwick et al. was used to estimate the orientation quaternions.

Regarding data collection methodologies, many of the above-mentioned gait analysis studies used body-mounted wearable sensors. There were a smaller number of studies using clothing mounted sensors in gait analysis; however, the results were promising [13,39,40]. Cleland et al. [13]. used a harness around the chest and waist with three sensors to compare step counting from body- and clothing-worn sensors Niazmand et al. [39] studied accelerometers mounted in ordinary trousers to identify freezing of gait in people with Parkinson’s, and their algorithm outputs were compared with a physician’s report. They reported an 88.3% sensitivity. Cha et al. [40] investigated the use of piezoelectric sensors mounted in loose trousers to recognise walking/periodic motion and concluded that it was possible to detect walking segments from the data at 93% accuracy compared to an algorithm developed by Cha et al. [41].

The present study aims to investigate how well the data from body-mounted sensors correlate with that from clothing-mounted sensors in terms of their orientation angles during walking and also what information can be extracted from multiple clothing-mounted sensors on trousers. de Jong et al. [15] studied sensors placed on the frontal and lateral sides of the upper shank during walking in terms of the shank-to-vertical angle, which is a parameter commonly used to describe orthosis alignment. Our study also involves sensors placed on the frontal (body) and lateral (clothing) sides, and we compare waist and thigh sensor pairs in addition to shank sensor pairs. Further, as in Lee et al.’s analysis, which presented visualisations of gait characteristics based on multiple joint angle information [18], we present the gait information in 3D plots incorporating information from multiple parts of the body, as well as using phase portraits.

## 3. Materials and Methodology

### 3.1. Materials

**Figure 1:**
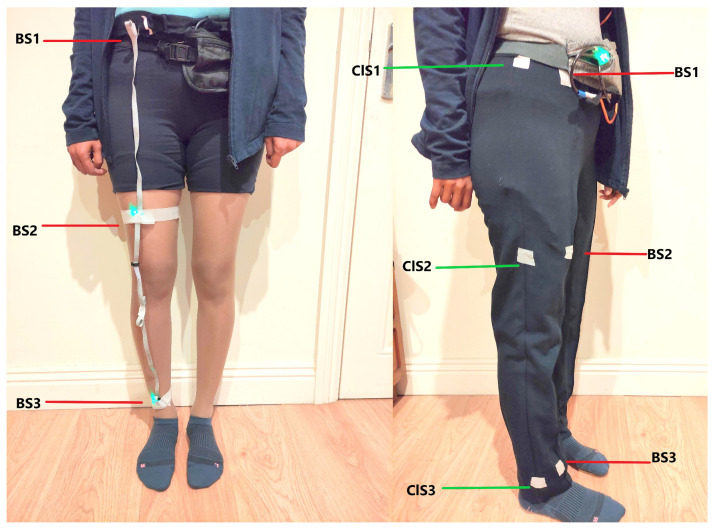
Sensor placement. On the body, there were three sensors on the frontal side of the body (BS1—waist, BS2—thigh, and BS3—lower-shank). On the clothing, there were three sensors along the lateral side of the lower body (ClS1—waist, ClS2—thigh and ClS3—lower-shank) in comparable positions to those on the body. Tape is used here to show the positions of the body-mounted and clothing-mounted sensors; however, in practice, neither was visible from the outside.

Our sensing system consisted of between 6 and 9 IMUs (based on the Bosch Sensortec BMI160 smart IMU) all using a synchronous bus and connected via flat ribbon cable to form a “sensor string”. Our in-house PCB design was fabricated by a commercial company at a cost of approximately 10 GBP per sensor. The bespoke sensors were approximately 15 × 12 × 7 mm each and had a combined weight of less than 14 g, and the inter-connecting cables weighed approximately 110 g. This sensor string was connected to a battery-powered Raspberry Pi, where the data were stored. The Pi and battery were worn in a waist pouch attached using a belt (Figure 1). Data were sampled at 50 Hz. The range of the accelerometers was ±16 g with a 12-bit resolution. The BMI160 IMU includes gyroscope, accelerometer and magnetometer readings.

Two sets of IMUs were worn by participants. One set was mounted inside the clothing, with three sensors along the lateral side of the lower body (waist, upper thigh and lower-shank) on the right side. The second set of IMUs was taped to the skin on the frontal side of the body at comparable positions to those of the sensors in the clothing, also on the right side (Figure 1). To attach the sensors to the clothing, the sensors were taped securely along the seams of the clothing in the chosen position, and cotton bias binding was taped on top of the sensor string using double-sided tape for fabric. In this way, the sensors were not outwardly visible and were also not in contact with the skin of the wearer. The body-mounted sensors were also part of the ‘sensor string’, and those sensors were encased in small woven pouches and taped to the skin/ body using microporous surgical tape to minimise the movements of the sensors.

### 3.2. Data Collection Procedure

Three healthy participants (age range: 35–36 years old; 2 males and 1 female) took part in this study. Each participant provided a pair of their own trousers in their usual size, and the researcher attached the sensors to the clothing. The male participants chose cotton-blend fleece jogging trousers, and the female participant chose loose cotton slacks. Even though both male participants wore jogging trousers, they were not of the same type. One participant’s trousers were baggy at the thigh, compared to the other participant’s. After placing/fixing the body-mounted sensor string, the participants were asked to put on the trousers with sensors and to connect the strings together to start the data collection.

Each day, the participants were asked to perform a set of predefined activities that included walking, with each activity contributing about 2 minutes’ data. The activities were: (1) standing still, (2) sitting on a chair, (3) 5 sit-to-stand and stand-to-sit cycles, (4) 5 leg raises, and (5) walking back and forth. These activities were video recorded to serve as a ground truth. Even though this analysis focuses only on walking data, ‘standing’, ‘sit-to stand’ and ‘leg raising’ data were also used for the sensor alignment as described in Section 3.3.1.

The study was reviewed by the research ethics committee of the School of Biological Sciences, University of Reading, UK and given a favourable ethical opinion for conduct (reference SBS 19- 20 31).

### 3.3. Data Analysis

We analysed the data from the walking activity from three participants over two days each. The data were first pre-processed (Section 3.3.1) and then segmented into individual gait cycles (Section 3.3.3). For each gait cycle, the sensor-to-vertical angles were estimated (Section 3.3.2) and used as the basis for comparing clothing-mounted with body-mounted sensors (Section 3.3.4).

#### 3.3.1. Pre-Processing

The data were collected in a compressed format on the Raspberry Pi and were transferred to a PC, decompressed and analysed in MATLAB.

As the initial orientations of the sensors relative to the limbs and to the world were not known, two rotation matrices were applied to each sensor dataset to transform the data into a common coordinate frame. A first rotation matrix was applied to align the z-axis of the sensor data with the direction of gravity. This rotation was derived from standing data, assuming that while the participant was ‘standing upright’, the limbs were all vertical and the only accelerations measured by the sensors were the accelerations due to gravity. The rotation was computed using Rodrigues’ rotation formula [42] by identifying the axis for rotation as being perpendicular to both the gravity vector and the z-axis.

**Figure 2:**
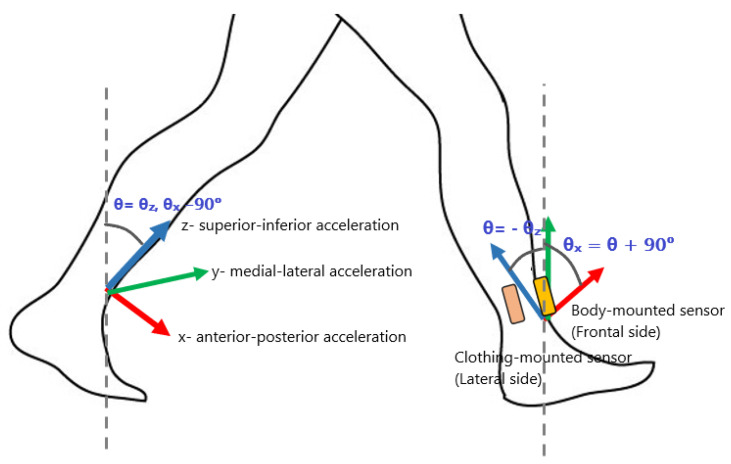
Location of sensors and corresponding coordinate frame {S}. Anterior-posterior accelerations of walking are measured in the sagittal plane and are principally measured by the x and z-axis in the sensor frame {S}. Angular velocities are principally measured by the y-axis of {S}. θ has a negative value when the leg is inclined (back leg, left) and a positive value when the leg is reclined (front leg, right).

A second rotation was applied to align the x-axis with the anterior-posterior direction in the sagittal plane and the y-axis with the medial-lateral direction perpendicular to the sagittal plane. The computation of the second rotation was based on finding the direction of forward-backward accelerations during movements that lie primarily within the sagittal plane, mentioned in Section 3.2 (e.g., sit-to stands, leg raising and walking). Figure 2 shows the alignment of the lower shank sensor. After this process, the clothing-mounted and body-mounted sensors can be assumed to be ‘axis’ aligned with the participant.

The accelerometer and gyroscope data were low-pass filtered using a second-order Butterworth filter with a 3 Hz cut-off. The filter was run on the data both forwards and backwards to minimise phase distortions.

#### 3.3.2. Sensor-To Vertical Angle Estimation

The primary accelerations of the leg and body segments during walking will be in or parallel to the sagittal plane with the rotational velocity vectors considered perpendicular to this plane. Following the pre-processing described in Section 3.3.1, it may be assumed that the sensor coordinate frame as shown in Figure 2 aligns so that the x and z-axis are contained in the sagittal plane. The y-axis is then aligned so as to measure the principal angular velocities of movement.

Inertial data from individual sensors placed on the clothing and attached to the skin was converted to quaternions using Madgwick’s algorithm [37] (https://github.com/xioTechnologies/NGIMU-Software-Public, accessed on 21 September 2021). The algorithm returns an estimate *q* of the quaternion values that relate the sensor coordinate frame {S} to a north (x-axis), west (y-axis), and up (z-axis) global frame {NWU}. These quaternions were assumed to be a reasonable estimate of the sensor frame {*S*} orientations in this study.

Computing the sensor-to-vertical angle through the gait cycle is now possible by considering any vector in the sensor frame ${}^{S}\vec{r}$ and computing its coordinates in the ${}^{\mathrm{NWU}}\vec{r}$ frame using

$${}^{\mathrm{NWU}}\vec{r}=q⊗{}^{S}\vec{r}⊗q^{*}$$

Thus the x-axis of the sensor frame (${}^{S}\vec{r}={\left[\begin{array}{ccc} 1 & 0 & 0 \end{array}\right]}^{T}$) and the z-axis (${}^{S}\vec{r}={\left[\begin{array}{ccc} 0 & 0 & 1 \end{array}\right]}^{T}$) have the respective coordinates

$$\begin{array}{ccc} {}^{\mathrm{NWU}}\vec{x}=\left[\begin{array}{c} {q_{0}}^{2}+{q_{1}}^{2}-{q_{2}}^{2}-{q_{3}}^{2}\\ 2q_{1}q_{2}-2q_{0}q_{3}\\ 2q_{0}q_{2}+2q_{1}q_{3} \end{array}\right]\; & \mathrm{and} & \;{}^{\mathrm{NWU}}\vec{z}=\left[\begin{array}{c} 2q_{1}q_{3}-2q_{0}q_{2}\\ 2q_{0}q_{1}+2q_{2}q_{3}\\ {q_{0}}^{2}-{q_{1}}^{2}-{q_{2}}^{2}+{q_{3}}^{2} \end{array}\right] \end{array}$$

Since we are only interested in the sensor-to-vertical angle measurement, only the last row of these two vectors is relevant, as they are the cosine of the angle between the sensor x-axis or z-axis and up. The remaining terms are only relevant for estimating the absolute orientation of the sagittal plane with respect to north.

Thus, the angle between the sensor x-axis and up is $\theta_{x}$, and the angle between the sensor z-axis and up is $\theta_{z}$, where

$$\begin{array}{cc} \theta_{x}= & \arccos(2q_{0}q_{2}+2q_{1}q_{3})\\ \theta_{z}= & \arccos({q_{0}}^{2}-{q_{1}}^{2}-{q_{2}}^{2}+{q_{3}}^{2}) \end{array}$$

It may seem logical to use the angle between the z-axis and up as the sensor-to-vertical angle; however, as this is an inverse cosine, it is not possible to determine if the limb is leaning forward or backwards with respect to up while walking. An alternative solution is to use the orientation of the x-axis with respect to up. During a normal gait cycle, this angle will oscillate around $90^{\circ }$ during walking, so an offset is subtracted from this angle, and the eventual calculation is

(1) $$\theta =\arccos\left(2q_{0}q_{2}+2q_{1}q_{3}\right)-90^{\circ }$$

The estimate of the angular velocity is simply that of the gyroscope y-axis, which, due to the preliminary processing, is also the axis perpendicular to the sagittal plane.

#### 3.3.3. Extraction of Gait Cycles

To segment the data into individual gait cycles, the MS points (right before the foot was at the terminal swing) were identified from the lower-shank accelerometry, following the method described in [11]. That is, for each of the datasets, the peaks above 2 g and 1.8 g within less than a 1 s window were identified from the acceleration magnitude from the clothing-mounted and body-mounted sensors, respectively. These peaks were logged as the MS points [11].

The duration of each individual gait cycle was then calculated as the time between two MS points. For each participant for each day, the mean durations for clothing and body sensors were calculated. The data were then segmented into individual gait cycles using the MS point as the start of each gait cycle, and setting the length equal to the mean duration in order that all gait cycles could have the same length (avoiding the need for normalisation). The data from the waist and thigh sensors, which were synchronised with the lower-shank sensors, could then be segmented into individual gait cycles using the same start and end points determined from the lower-shank sensor.

This process of gait cycle extraction was performed programmatically, and to check the accuracy of the algorithm, the start and end points of each gait cycle were identified from the video ground truth and checked against the points that had been programatically identified.

For each gait cycle, initial contact (IC) and toe off (TO) points were identified as the local minima in the lower-shank gyroscope data around the medial-lateral axis (y-axis), as used by Tjhai and O’Keefe [9].

#### 3.3.4. Comparison of the Body-Mounted and Clothing-Mounted Sensor Angles

After verifying the normality of the data [43], the correlation (Pearson’s correlation coefficient) between the sensor-to-vertical angles of the clothing- and body-mounted sensors [44] was calculated for each individual gait cycle. In total, 465 gait cycles were analysed from the 3 participants (i.e., 465 correlation coefficients). The mean correlation coefficient value was calculated for each participant.

The difference between the angles of each sensor pair (subtracting the body-mounted sensor angle from clothing-mounted angle) was also calculated for each participant for 2 min of standing and at two points in the gait cycle. During standing, the angle differences were expected to be minimal, as the orientation correction performed during pre-processing (Section 3.3.1) should ensure that the data from both sensors were aligned with vertical.

For walking, the angle differences were calculated at two different points. One value was calculated at the IC point extracted based on the lower-shank sensor, with the rationale that the difference between the two sensors could potentially be greatest at this point in the gait cycle. A second value was calculated at the point where the lower-shank sensor on the body was vertical during stance phase, with the rationale that the difference between the two sensors could potentially be smallest at this point in the gait cycle. The angle differences between the body-mounted and the clothing-mounted sensors was calculated for each gait cycle at these two points, and the mean and standard deviation across all gait cycles was calculated for each participant.

## 4. Results

### 4.1. Extracted Gait Cycles

**Figure 3:**
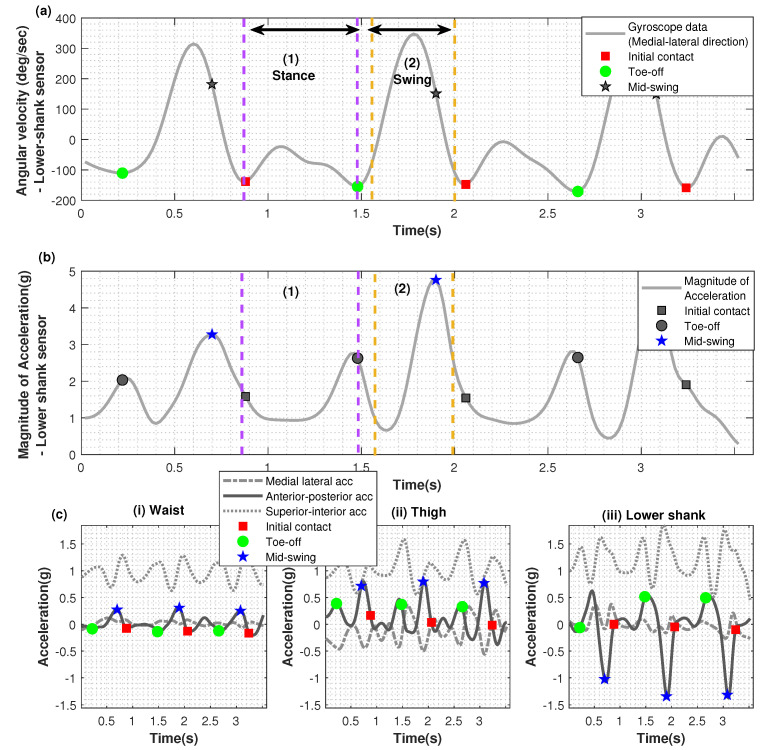
Data from the clothing-mounted sensors for participant 1, day 2. Initial contact and toe-off markers were extracted based on the gyroscope data (medial-lateral axis) from the lower shank (**a**), and the mid-swing markers were extracted based on the magnitude of the accelerometer data from the lower shank (**b**). In (**c**), the same timepoints are shown on the anterior-posterior accelerations for the i. waist, ii. thigh, and iii. lower shank. Segment (1) bounded by purple dashed lines indicates the ‘stance’ phase, and segment (2) bounded by yellow dashed lines indicates the ‘swing’ phase.

Figure 3a–c shows the data from the clothing-mounted sensor on the lower-shank for two gait cycles of one particpant, along with the extracted initial contact (IC), toe-off (TO) and mid-swing (MS) points. The local minima for extracting the IC and TO points could be clearly identified in the gyroscope data from the clothing-mounted sensor (Figure 3a). Similarly, the local maxima for extracting the MS points could be clearly identified from the accelerometer data of the clothing-mounted sensor ((Figure 3b). The same points (IC, TO and MS) marked on the waist, thigh and lower-shank acceleration data are shown in Figure 3(c.i–c.iii). Approximate MS points were extracted by using the peak values marked on Figure 3b based on the magnitude of lower-shank/ankle accelerometer data. Comparing with the MS points that were identified manually from the video, the programmatic approach correctly identified the points from the clothing-mounted sensor data with 97.9% accuracy, and at an accuracy of 99.7% in the body-mounted sensor data. Inaccuracies seemed to have occurred in the clothing data when some ‘turning’ data were identified as MS points by the algorithm. The analysis presented in the following sections considers only the gait cycles with correctly identified MS points.

### 4.2. Sensor-to-Vertical Angles

**Figure 4:**
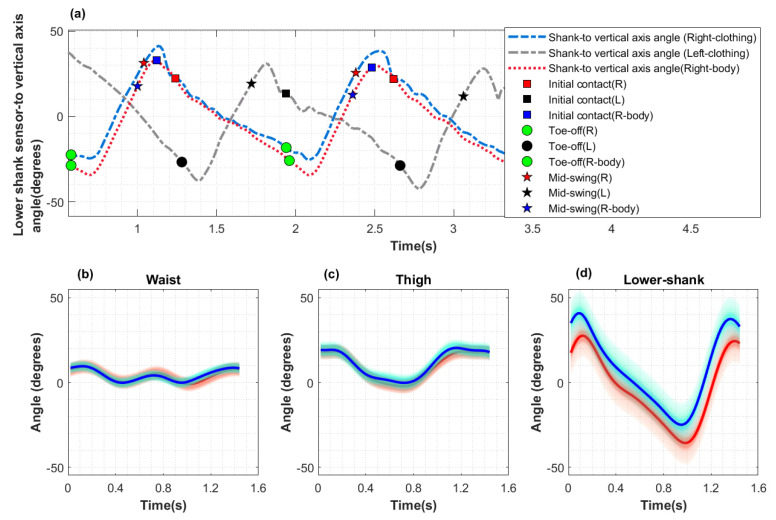
Body- vs. clothing-mounted sensor-to-vertical angle for Participant 2, day 2. (**a**) Exemplar initial contact (IC), toe-off (TO), and mid-swing (MS) markers on the lower-shank sensor data of the body (right leg) and clothing (left and right legs). (**b**–**d**) Mean gait cycles across 67 gait cycles for body-mounted (red) and clothing-mounted (blue) sensors for the (**b**) waist, (**c**) thigh, and (**d**) lower-shank sensors; shaded areas represent the standard deviation.

The sensor-to-vertical angles for the body- (right side) and clothing-mounted (left and right sides) lower-shank sensors for Participant 2, day 1 are shown in Figure 4a. These lower-shank angles were comparable with the shank-to vertical angle analyses presented by Gujarathi and Bhole [30] and with the inverse angle values presented by de Jong et al. [15]. The signs for the present study are inverted to those of de Jong et al., as the authors used positive angles to represent when the leg was inclined and negative angles when the leg was reclined, while the convention was the other way around in the present study.

As described in Section 3.3.3, MS points extracted from the lower-shank acceleration data were used as the starting point to also segment the gait cycles from the waist and thigh sensors. In this study, 465 gait cycles were analysed across all participants. The mean for 67 gait cycles from one participant, for both body-mounted and clothing-mounted sensors, is shown in Figure 4b–d.

### 4.3. Waist, Thigh, and Lower-Shank Sensor-to-Vertical Angles

**Figure 5:**
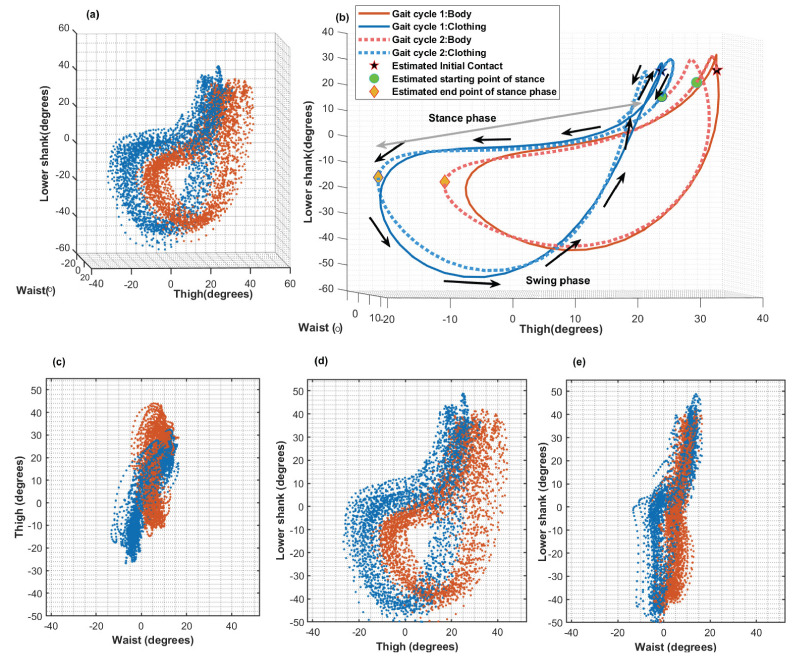
Sensor-to-vertical angles of the waist, thigh, and lower shank plotted against each other for 53 gait cycles from Participant 2, day 1. (**a**) The angles for body-mounted sensors (red dots) and clothing-mounted sensors (blue dots) plotted in a 3D space. (**b**) Two typical gait cycles from body-mounted (red) and clothing-mounted (blue) sensors. ‘Green o’ s are the approximate starting points of stance phases (Initial contact) and ‘yellow diamonds’ are the approximate end points of swing phases (toe off). The arrows show the direction of angle changes over the course of a gait cycle. (**c**–**e**) represent the angle data for ‘thigh’ vs. ‘waist’, ‘lower shank’ vs. ‘thigh’ and ‘lower shank’ vs. ‘waist’, respectively.

Figure 5 shows the sensor-to-vertical angles of the waist, thigh, and lower shank plotted against each other over the course of multiple gait cycles. Both the data for the body-mounted sensors (red) and for the clothing-mounted sensors (blue) are shown. Visual examination shows that both datasets follow a similar shape with a slight offset. The amount of offset may vary with clothing material. In this particular dataset, Participant 2 was wearing loose slacks, and hence, the lower-shank sensor might experience movement from the clothing in addition to the leg movement.

The mean angle differences between the clothing and body sensors are shown in Table 1. It can be noted that Participant 2’s mean angle difference (row 2, column 9) at the IC stage was the highest compared to the other two participants.

**Figure 6:**
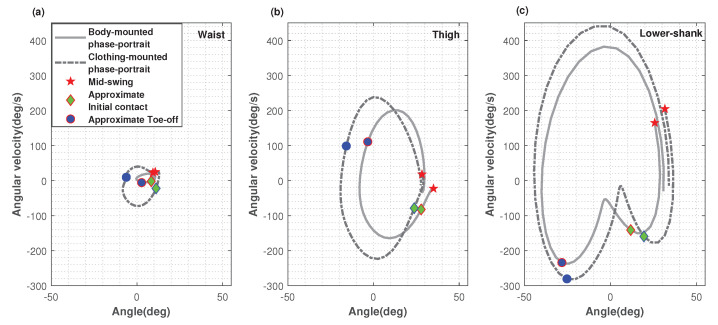
Phase portraits for a typical gait cycle from body-mounted and clothing-mounted sensor pairs from Participant 2, day 1. The angular velocity is plotted against the sensor-to-vertical angle for the (**a**) waist sensor pair, (**b**) thigh sensor pair and (**c**) lower-shank sensor pair. ‘Red *’s denote the starting points (mid-swing, MS), ‘green and cyan diamonds’ denote approximate initial contact (IC) and ‘blue o’ s denote approximate toe-off (TO) for each cycle. These plots indicate that clothing-mounted sensors had a wider range of angle changes and angular velocity changes than the body-mounted sensors.

Figure 6 illustrates how the angular velocity changes against the sensor-to vertical angle over a gait cycle for each sensor by using phase portraits. The figure shows a typical gait cycle which follows the shape of the mean gait cycle, as shown in Figure 4. Approximate MS, IC and TO points are marked on each plot for body-mounted and clothing-mounted sensors. This shows that the clothing-mounted sensor data experienced a wider range of angles in walking, while having a similar body-mounted gait cycle shape in the phase portrait. Further, it could be noted that the MS, IC and TO points identified from clothing and body mounted sensors were close to each other (pair-wise).

### 4.4. Correlation Coefficient Analysis

**Figure 7:**
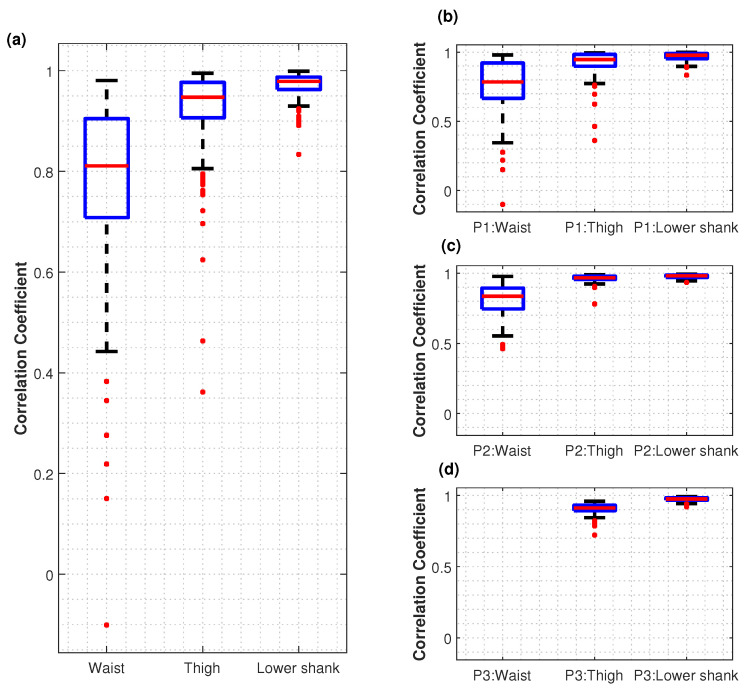
Correlations between the body-mounted sensor vs. clothing-mounted sensor in terms of their sensor-to-vertical angles over the course of a gait cycle. The plots show Pearson correlation coefficient values for (**a**) all 465 gait cycles, (**b**) Participant 1’s data, (**c**) Participant 2’s data and (**d**) Participant 3’s data. P3 did not have a body-mounted ‘waist’ sensor.

The distributions of correlation coefficients comparing the sensor-to-vertical angle over individual gait cycles for body-mounted sensors vs. clothing-mounted sensors is shown in Figure 7. The box plots in Figure 7a indicate that the ‘lower shank’ and ‘thigh’ sensor pairs maintained a higher correlation than the ‘waist’ sensor pairs.

The mean correlation coefficient values are reported in Table 1. Values ranged from 0.97–0.98 for the lower shank and from 0.91–0.96 for the thigh, whereas the values for the waist were lower at 0.77 and 0.81.

In addition, there were notable outliers in the ‘waist’ sensor data from ‘Participant 1’ (Figure 7b).

## 5. Discussion

The main intention of this study was to examine how well data from multiple, frontal body-mounted sensors correlate with the data from lateral side clothing-mounted sensors with respect to ‘walking’ data and to find whether key gait related information can be extracted from the clothing-mounted sensors. From the overall analysis, it was possible to observe that even though the clothing data were not in full agreement with the body-mounted sensor readings, clothing data could be used to estimate and track useful gait information such as the IC, TO and MS points (Figure 3). Further, Figure 4, which shows the sensor-to-vertical angles for the left and right clothing-mounted sensors and for the right side body-mounted sensor on the lower-shank, shows that the lower shank gait information related to gait cycles are in close proximity in both data streams. Moreover, by examining Figure 4, it was noted that IC points from one leg (blue ‘square’) were approximately aligned with TO (black ‘o’) points on the other leg.

By simultaneously observing the angle changes in the waist, thigh and lower-shank sensors throughout the gait cycle as shown in Figure 4b–d and Figure 5, the whole leg movement can be captured by the three angles, which would not be possible with only a single sensor. Further, having the angles of the waist, thigh and lower-shank angles together can help with visualising the trunk, thigh and tibia movements, respectively. As Gao et al. explained [21], the angle information from the three sensors could potentially be sufficient for activity classification without the need for multiple heuristic features.

Figure 6c indicates that from IC to TO, changes of angular velocity seem to decrease slowly, while the angle along the vertical axis was being changed. This implies that this leg (right) was on the floor and the other leg (left) might be in swing phase, moving the body, which caused only the angle changes in the right leg. Figure 6b,c show quite similar changes in both sensors on body and clothing at stance phase (from ‘green diamond’ to ‘blue o’). This pattern was noticed with all the other datasets as well. However, Figure 6a shows a slightly magnified shape of clothing-mounted phase portrait for body-mounted waist phase portrait. With some of the datasets, this incident was noted in the other direction, having a magnified phase portrait for clothing-mounted sensors.

From Table 1, it was noted that the body-mounted and clothing-mounted sensors had a mean angle difference of around $0^{\circ }$ while the participants were standing, and it can be said that at the beginning of the data collection, the sensor pairs were at more or less similar orientations. Further, angle differences at the ‘shank vertical’ stage were comparatively lower than those at IC. This may have occurred as, at the ‘shank vertical’ stage, the clothing-mounted sensors were nearly at a resting state compared to the IC point, where there was an additional acceleration of the clothing.

There were also differences in the angle differences across participants, possibly due to differences in the clothing material and the fit of the clothing. For example, at IC, we saw larger angle differences at the lower shank for Participant 2, who was wearing loose cotton slacks, as compared with Participants 1 and 3, who were wearing jogging trousers with elastic at the ankles. Additionally, Participant 1’s trousers were baggy at the thigh compared with Participant 2’s, which could account for the larger angle difference observed in Participant 1’s data at the thigh as compared with Participant 2’s. We also noted that the orientation of the lower-shank sensors in the clothing could be affected by whether or not the participants were wearing shoes, and so putting on or removing shoes midway through data collection would affect the alignment between the body- and clothing-mounted sensors.

Figure 4, Figure 5 and Figure 6 also indicated that clothing-mounted sensor data have a higher range of angle values than that of body-mounted sensor data. By observing the mean gait cycle shapes in Figure 4d–d and Figure 5b with respect to both sensors (body-mounted and clothing-mounted), it can be further noted that clothing-mounted cycles had the same shape with an amplitude of data comparable to body-mounted data. Figure 5a shows the regularity of the walking pattern of this participant. Figure 5c–e also illustrates that each sensor pair had a similar shape with a higher range of values in clothing sensors. Figure 5b was drawn by taking into account the mean gait cycles from body- and clothing-mounted sensor data. Approximate IC and TO points for cycles were marked on the mean gait cycles to examine the stance and swing phases. Those angle variations could have happened due to looseness of clothing, as this can add additional movements, especially at the ‘thigh’ and ‘lower shank’ points when the person was walking with a higher acceleration. Even though an orientation correction mechanism was applied to the sensor data based on standing and walking segments (as mentioned in Section 3.3.1), sensors on clothing may have altered their positions.

However, Pearson’s correlation coefficient analysis allowed interpretation of the data in a different way. Mean correlation coefficient values (Table 1) revealed that the waist sensor pair had the least correlation among all the participants, whereas lower shank and thigh had higher correlation values of more than 0.97. Yet, the waist sensor pairs also had a correlation above 0.76. These correlation coefficient values were further analysed based on the box plots shown in Figure 7. It was recognised that there were a few outlier values in waist sensor pairs. When examining the outlier data, it was noted that, at the point where a gait cycle occurred with a negative correlation coefficient value, both body-mounted and clothing-mounted signals deviated from the mean ‘waist’ gait cycle. However, the other two sensors had maintained the correlation at a higher level at that specific point. As both ‘waist’ signals had been changed at that point, it is possible that due to a hand movement or due to the band of the ‘waist pouch’, a sudden displacement of the sensors might have happened. Examining other outlier points, it was noted that clothing-mounted sensor data agreed with the mean gait cycle, while the body-mounted sensor data deviated from the mean gait cycle. This can be justified as the body-mounted sensor position might change slightly owing to the movements of the waist band of the trousers and the waist pouch because the trousers were worn nearly on top of the body-mounted waist sensor. Keeping the ‘waist’ sensor on the waist line of the trousers and waist-pouch band around the waist might have added additional movements or prevented the movements of the waist sensor. This may have caused the slightly lower correlation coefficient with respect to the sensor-to vertical axis angle than that of ‘lower shank’ and ‘thigh’ data. Hence, although the clothing-mounted sensors may not be a perfect representation of gait, the same can be said with the body-mounted sensors. Clothing- and body-mounted sensors have similar information content when considering gait (Figure 3a,b).

However, if the clothing is excessively loose or if the sensor is disoriented after doing the alignment, the results would not be as accurate as the expected results. However, even with that kind of subtle misalignment in the middle of the data collection, data would be able to give gait characteristics in a reasonable way (Figure 5). Wearing trousers that are not too loose-fitting near the lower shank/ankle and attaching the sensors firmly to the fabric to firmly fix their orientation would minimise these issues.

Future work includes using clothing-mounted sensor data in activity classification, using the structure of the gait as the feature that may improve the classification accuracy. Assuming that the clothing-mounted sensors are a feasible way of collecting data from people who have mobility disorders in an unobtrusive way, data could be collected for a longer period to analyse how gait patterns change due to factors such as fatigue, gait changes over extended periods of time, or changes of gait in response to a pharmaceutical, surgical or rehabilitative intervention.

## 6. Conclusions

In this study, we have collected and analysed data from multiple lightweight, time-synchronised sensors mounted into everyday loose clothing. Even though the data from clothing-mounted sensors showed a larger range of angle variation compared to that from the body-mounted sensors, the sensor pairs correlate well at key points in the gait cycle. The results also indicate that the data from the clothing-mounted sensors can be used in extracting and analysing the gait cycles in a productive way. Hence, we conclude that sensors mounted in loose clothing are a promising way of studying human movement.

## Figures and Tables

**Table 1:** Mean correlation coefficient (corr. coef.) between each sensor pair and mean angle differences (subtracting body-mounted sensor angle from clothing-mounted angle) with standard deviation values (denoted with ±). For standing, the angle differences were calculated over 2 min of data. For walking, the angle differences were calculated at initial contact (IC) and at the point when the lower-shank body-mounted sensor was approximately vertical. P3 did not have a waist sensor pair (indicated by ‘-’).

	Clothing Type	Waist				Thigh				Lower Shank			
		Corr. Coef.	Standing	Walking IC	Walking Shank Vertical	Corr. Coef.	Standing	Walking IC	Walking Shank Vertical	Corr. Coef.	Standing	Walking IC	Walking Shank Vertical
**P1**	Jogging trousers	0.77	$0.03^{\circ }\pm$ $0.52^{\circ }$	$-1.95^{\circ }\pm$ $6.94^{\circ }$	$2.11^{\circ }\pm$ $4.30^{\circ }$	0.93	$0.06^{\circ }\pm$ $0.30^{\circ }$	$-14.57^{\circ }\pm$ $8.47^{\circ }$	$-8.82^{\circ }\pm$ $8.13^{\circ }$	0.97	$0.14^{\circ }\pm$ $0.37^{\circ }$	$2.12^{\circ }\pm$ $7.55^{\circ }$	$2.83^{\circ }\pm$ $5.87^{\circ }$
**P2**	Loose slack	0.81	$0.13^{\circ }\pm$ $0.34^{\circ }$	$0.85^{\circ }\pm$ $2.45^{\circ }$	$-1.60^{\circ }\pm$ $2.83^{\circ }$	0.96	$0.01^{\circ }\pm$ $0.28^{\circ }$	$-2.91^{\circ }\pm$ $7.60^{\circ }$	$-4.00^{\circ }\pm$ $7.23^{\circ }$	0.98	$0.03^{\circ }\pm$ $0.23^{\circ }$	$11.16^{\circ }\pm$ $7.72^{\circ }$	$7.20^{\circ }\pm$ $4.06^{\circ }$
**P3**	Jogging trousers	-	-	-	-	0.91	$0.12^{\circ }\pm$ $0.30^{\circ }$	$-6.27^{\circ }\pm$ $9.71^{\circ }$	$-2.21^{\circ }\pm$ $3.62^{\circ }$	0.97	$-0.07^{\circ }\pm$ $0.30^{\circ }$	$4.47^{\circ }\pm$ $8.88^{\circ }$	$3.59^{\circ }\pm$ $4.76^{\circ }$

## Data Availability

Data supporting the results of this study are available within the article and its Supplementary Materials.

## Supplementary Materials

The following are available online at https://www.mdpi.com/article/10.3390/s22176605/s1.
